# Breakthrough Infection After a Primary Series of COVID-19 Vaccination Induces Stronger Humoral Immunity and Equivalent Cellular Immunity to the Spike Protein Compared with Booster Shots

**DOI:** 10.3390/vaccines13070751

**Published:** 2025-07-13

**Authors:** Yoshifumi Uwamino, Takashi Yokoyama, Yasunori Sato, Shiho Tanaka, Yuka Kamoshita, Ayako Shibata, Toshinobu Kurafuji, Akiko Tanabe, Tomoko Arai, Akemi Ohno, Ho Namkoong, Tomoyasu Nishimura, Masatoshi Wakui, Mitsuru Murata, Naoki Hasegawa, Hiromichi Matsushita

**Affiliations:** 1 Department of Laboratory Medicine, Keio University School of Medicine, 35 Shinanomachi, Shinjuku-ku 160-8582, Tokyo, Japan; 2 Department of Infectious Diseases, Keio University School of Medicine, 35 Shinanomachi, Shinjuku-ku 160-8582, Tokyo, Japan; 3 Department of Biostatistics, Keio University School of Medicine, 35 Shinanomachi, Shinjuku-ku 160-8582, Tokyo, Japan; 4 Clinical Laboratory, Keio University Hospital, 35 Shinanomachi, Shinjuku-ku 160-8582, Tokyo, Japan; 5 Keio University Health Center, 35 Shinanomachi, Shinjuku-ku 160-8582, Tokyo, Japan; 6 Research Center for Clinical Medicine, International University of Health and Welfare, 4-1-26 Akasaka, Minato-ku 107-8482, Tokyo, Japan

**Keywords:** booster effect, BNT162b2 vaccine, breakthrough infection, cellular immunity, humoral immunity, immune response

## Abstract

**Background:** The long-term immune implications of administering more than four doses of COVID-19 vaccine and the impact of breakthrough infections are not fully understood. **Research Design and Methods:** We conducted a follow-up cohort study on Japanese healthcare workers who received more than three doses of the BNT162b2 vaccine. We assessed both the anti-SARS-CoV-2 antibody titer and cellular immunity in 429 participants and investigated the numbers, types, and brands of COVID-19 vaccines administered, as well as the episodes of COVID-19 infections after the third dose. **Results:** Individuals who received three total doses of vaccines with BTI episodes demonstrated higher antibody titers than those who received four total doses of vaccines with no BTIs. The cellular immune responses between these two groups were comparable. **Conclusions:** These findings suggest that BTIs occurring after the primary series of COVID-19 vaccinations (first to third dose) induced humoral immunity to the spike protein that is greater than that induced by booster doses (fourth or fifth dose) and elicit cellular immunity to the spike protein comparable to that of booster doses.

## 1. Introduction

The coronavirus disease 2019 (COVID-19) pandemic, caused by severe acute respiratory syndrome coronavirus 2 (SARS-CoV-2), has had a profound global impact on public health, leading to significant social and economic disruption. To mitigate the spread of the virus and the severity of the disease, numerous highly effective vaccines have been developed [1,2]. However, the emergence of mutant strains has resulted in recurring waves of infections. The Omicron strain has shown immunological escape. The originally developed monovalent vaccines for wild-type strains demonstrated limited protection against the Omicron strain infections [3], while robust protection against life-threatening infections remained [4]. To optimize the immunity for circulating variants at the time, bivalent vaccines targeting both wild type and Omicron BA1 or BA4/5 were developed [5]. Booster shots can lead to elevated levels of immunoglobulins, indicating short-term benefits in enhancing immune response [6]. Nevertheless, the long-term immune implications of administering four or more doses of the vaccine remain poorly understood.

In Japan, the third dose of monovalent mRNA vaccines (BNT162b2 and mRNA-1273), which targets wild type strains, was recommended for the general population from December 2021 to August 2022. Additionally, a fourth dose of monovalent mRNA vaccine was offered to older adults and healthcare professionals from May 2022 to early September 2022. As of late September 2022, the vaccination strategy transitioned to bivalent vaccines (wild type and Omicron BA1 or BA4/5). Since then, all adults were recommended to receive one dose of bivalent vaccine. The minimum interval between each booster dose was 3 months. Therefore, as of January 2023, medical professionals had the opportunity to receive a maximum of three booster doses (the third, fourth, and fifth dose) of COVID vaccines within approximately 1 year from December 2021.

Nonetheless, knowledge regarding the effects of multiple booster vaccinations on long-term humoral and cellular immunity remains limited. Additionally, since the outbreak of the Omicron variant, Japan has experienced a surge in COVID-19 cases, accompanied by a significant number of breakthrough infections (BTIs). Although some reports have suggested that BTI may provide a booster effect on humoral immunity comparable to that of booster vaccinations [7], data on the impact of BTI on cellular immunity are still scarce.

In February 2021, we initiated a prospective cohort study to analyze humoral and cell-mediated immunity to the spike protein (S protein) in 673 healthy healthcare workers who received primary series of the BNT162b2 monovalent vaccine during a mass vaccination campaign (Clinical Trial Registration: www.umin.ac.jp/ctr identifier is UMIN000043340) [8]. In the present follow-up study, we aimed to disclose the relationship between number of doses, vaccine types, and immunity in the healthy adults with or without BTI.

## 2. Patients and Methods

### 2.1. Study Participants

This study was conducted as part of a prospective cohort study evaluating the effectiveness of COVID-19 vaccines among healthy healthcare workers at Keio University Shinanomachi Campus (Tokyo, Japan). The cohort included 673 healthcare workers who received two doses of the BNT162b2 monovalent vaccine (Pfizer, New York, NY, USA) between February and April 2021. Most of the cohort participants received a third dose of the BNT162b2 monovalent vaccine at Keio University Hospital (Tokyo, Japan) in December 2021.

To evaluate humoral and cellular immunity approximately one year after the third dose, as well as the effects of the fourth and fifth doses received in the interim and the association with BTI occurring post-third dose, serum and whole blood samples were collected between 26 January and 9 February 2023. The decision to receive the fourth and fifth doses was left to the discretion of each individual. Participants who opted for additional doses received them at vaccination sites run by government agencies or nearby medical facilities at their chosen intervals. The vaccine brand (BNT162b2 or mRNA-1273 [Moderna, Inc., Cambridge, MA, USA]) and whether it was a monovalent or bivalent vaccine (vaccine type) varied depending on the administering medical institution. At the same time as sample collection, data regarding the history of BTI between the date of third-dose administration (December 2021) and the date of sample collection (during the period, Omicron BA1, BA4, and BA5 were dominant variants in Japan [9]), the total number of vaccine doses received, the date of vaccination, the type, and the brand of the vaccine used as the fourth and fifth doses, as well as information on adverse reactions, were obtained using a web-based questionnaire. Additionally, the date of BTI (the date of symptoms starting for symptomatic individuals and the date of a positive test for asymptomatic individuals) were obtained from the health records of employees of the Keio University Shinanomachi Campus and from electronic medical records of Keio University Hospital, where the occupational physician performed SARS-CoV-2 detection tests and COVID-19 diagnosis.

From the participants who received 3 doses of BNT162b2 monovalent vaccines until January 2022, those who completed both sample collection and responded to questionnaires were included in the analysis. Those participants who declared they had experienced a BTI after the administration of the third dose but whose BTI episodes were not recorded were excluded from the analysis. Furthermore, to eliminate the impact of SARS-CoV-2 infection before the third dose (i.e., before Omicron variants became dominant in Japan), cases with BTI before the third dose were excluded from the analysis, these cases were detected by the conversion to seropositivity of nucleocapsid protein IgG antibody [8]. Additionally, the participants who experienced more than 2 episodes of BTI after the third dose were also excluded to evaluate the impact of single BTI episode as compared to that of the booster dose. This study was approved by the Ethics Committee of Keio University School of Medicine (20200330). Written informed consent was obtained from all participants.

### 2.2. Measurement of Immunity

Anti-receptor binding domain (RBD) IgG antibody (RBD-IgG) was detected using SARS-CoV-2 IgG II Quant reagents (Abbott Laboratories, Abbott Park, IL, USA), and anti-Omicron neutralizing antibodies (NA-BA5) were detected with authentic Omicron BA5 SARS-CoV-2 virus (TA41-702, provided by the National Institute of Infectious Diseases, Japan), following a previously described method [10]. Furthermore, an interferon γ-releasing assay to assess cellular immunity was performed using QuantiFERON SARS-CoV-2 reagents (QFN; Qiagen, Hilden, Germany). Interferon γ levels were measured after exposing the samples to Antigen 1 (a spike protein-specific long-chain peptide) and Antigen 2 (an RBD-specific short-chain peptide), and the values were normalized by subtracting the negative control values (QFN-Ag1 and QFN-Qg2). The details of the assays have been described previously [8]. Any extremely high or low interferon γ levels (i.e., higher than the positive control or lower than the negative control) were recorded, and the data were excluded from the analysis.

### 2.3. Statistical Analysis

The antibody titers and cellular immunity data were transformed by logarithmization to approximate a normal distribution. Therefore, the evaluation was conducted using the log values (base 10) of RBD-IgG, NA-BA5, QFN-Ag1, and QFN-Ag2 (Log_10_RBD-IgG, Log_10_NA-BA5, Log_10_QFN-Ag1, and Log_10_QFN-Ag2, respectively). The medians of the Log_10_RBD-IgG, Log_10_NA-BA5, Log_10_QFN-Ag1, and Log_10_QFN-Ag2 were compared based on the BTI, and on the vaccine dose, type, and brand. Each category was defined as follows: BTI, episode(s) of BTIs after the administration of the third dose, vaccine dose, cumulative numbers of COVID-19 vaccine doses, vaccine type, administration of bivalent vaccines as a fourth and/or fifth dose, and administration of mRNA-1273 vaccines as a fourth and/or fifth dose.

Additionally, the participants were categorized into 6 groups according to the vaccine dose and BTIs as follows: 3 doses without BTI (3 doses/BTI(−)), 3 doses with BTI (3 doses/BTI(+)), 4 doses without BTI (4 doses/BTI(−)), 4 doses with BTI (4 doses/BTI(+)), 5 doses without BTI (5 doses/BTI(−)), and 5 doses with BTI (5 doses/BTI(+)). The median of Log_10_RBD-IgG, Log_10_NA-BA5, Log_10_QFN-Ag1, and Log_10_QFN-Ag2 were determined according to the groups. We compared Log_10_RBD-IgG, Log_10_NA-BA5, Log_10_QFN-Ag1, and Log_10_QFN-Ag2 between groups with and without BTI, for the same number of vaccine doses. Additionally, we examined the differences between groups with one additional vaccine dose but without BTI, and between groups with one more vaccine dose but no BTI compared to groups with fewer vaccine doses but with BTI. This implies a comparison of the effects of vaccines and BTIs when considering one vaccine dose and one BTI as equivalent antigen exposures by evaluating the same number of antigen exposures.

Comparisons of continuous variables were performed using the Mann–Whitney *U* test, and comparisons of categorical variables were conducted using Fisher’s exact test; *p*-values under 0.05 were considered as statistically significant.

To evaluate whether BTI episodes confer immunity comparable to that of vaccination, independent of age, sex, and time since the last antigen exposure, we performed ANCOVA model analyses for Log_10_RBD-IgG, Log_10_NA-BA5, Log_10_QFN-Ag1, and Log_10_QFN-Ag2 in participants with 3 doses/BTI(+) versus those with 4 doses/BTI(−) and 4 doses/BTI(+) versus those with 5 doses/BTI(−). These models incorporated two continuous variables: age and intervals from the last antigen exposure (duration between the sample collection date and BTI episode in BTI(+) groups or the last dose of vaccine administration in BTI(−) groups), as well as two categorical variables: sex and group (3 doses/BTI(+) vs. 4 doses/BTI(−) or 4 doses/BTI(+) vs. 5 doses/BTI(−)).

All statistical analyses were performed using JMP Pro, v.17 (SAS Institute, Cary, NC, USA) and Graph Pad Prism, v.9.0 software (Graph Pad Software, San Diego, CA, USA).

## 3. Results

Of the 554 participants who received three doses of BNT162b2 monovalent vaccines until January 2022, 447 participants cooperated with both sample collection and questionnaire completion. Of these, nine participants who answered that they had a history of BTI after third-dose administration but whose BTI episodes were not recorded were excluded. Additionally, five participants with BTI before the third dose and four participants with two BTI episodes after the third dose were excluded from the analysis. Therefore, a total of 429 participants were included in the analysis. Demographic characteristics of these participants according to the cumulative doses of vaccines and episodes of BTI are presented in Table 1.

There was a tendency for older participants to have received a higher number of vaccinations, whereas no sex differences were observed regarding the number of vaccinations.

No significant age differences were found in relation to the presence or absence of BTIs across participants with the same number of vaccinations. Apart from a significantly higher proportion of females in the 3 doses/BTI(+) group compared to the 3 doses/BTI(−) group (*p* = 0.0248), no sex-specific differences were observed among those with the same number of vaccinations. As expected, the greater the number of vaccinations, the shorter the elapsed time since the last vaccination. When comparing the 3 doses/BTI(+) group to the 4 doses/BTI(−) group, no sex-based differences were observed (*p* = 0.3806), but age in the 3 doses/BTI(+) group was significantly younger than that in the 4 doses/BTI(−) group (*p* = 0.0073). Intervals from the last antigen exposure were longer in the 3 doses/BTI(+) group than in the 4 doses/BTI(−) group (*p* < 0.0001).

**Figure 1:**
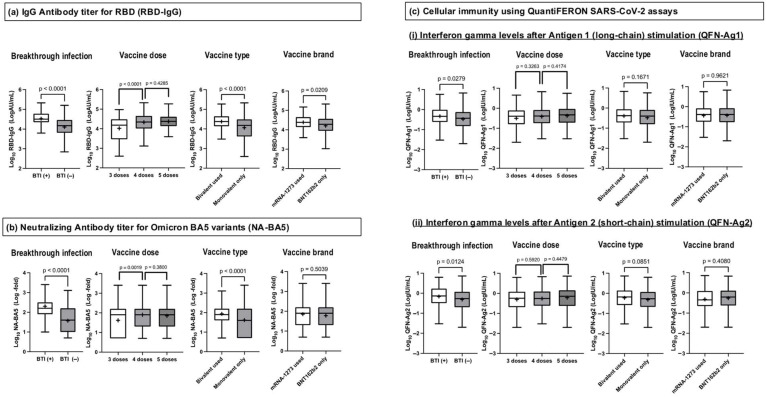
Humoral and cellular immunity at approximately 1 year after the third dose in different doses, vaccine brands, and types and relation to BTI. The bars show the medians of the logarithm values for (**a**) RBD-IgG, (**b**) NA-BA5, and (**c**) QFN-Ag1 and -Ag2 (Log_10_RBD-IgG, Log_10_NA-BA5, Log_10_QFN-Ag1, and Log_10_QFN-Ag2); the boxes show the quartile range; and the error bars show the maximum and minimum after the exclusion of outliers. “+” signs demonstrate the mean of Log_10_RBD-IgG, Log_10_NA-BA5, Log_10_QFN-Ag1, and Log_10_QFN-Ag2. Log_10_RBD-IgG, Log_10_NA-BA5, Log_10_QFN-Ag1, and Log_10_QFN-Ag2 were compared based on BTI, vaccine dose, type, and brand. Each category was defined as follows: BTI, episode of BTI after third-dose administration, vaccine dose, cumulative numbers of COVID-19 vaccine doses, vaccine type, administration of bivalent vaccines as fourth and/or fifth doses, vaccine brand, and administration of mRNA-1273 vaccines as fourth and/or fifth doses. Abbreviations: BTI, breakthrough infections after third-dose administration; RBD-IgG, IgG antibody titer specific to the receptor-binding domain of SARS-CoV-2 measured by chemiluminescent immunoassay; NA-BA5, neutralizing antibody titer measured using the authentic SARS-CoV-2 Omicron BA. Five variants: QFN-Ag1 and -Ag2; interferon gamma levels were measured using QuantiFERON SARS-CoV-2 assays. -Ag1 represents long-chain protein (Antigen 1)-stimulated results, and -Ag2 represents short-chain protein (Antigen 2)-stimulated results.

Figure 1 compares Log_10_RBD-IgG, Log_10_NA-BA5, Log_10_QFN-Ag1, and Log_10_QFN-Ag2 for the presence or absence of BTI, the number of vaccine doses, vaccine type, and vaccine brand. In participants where BTI was present, Log_10_RBD-IgG, Log_10_NA-BA5, Log_10_QFN-Ag1, and Log_10_QFN-Ag2 levels were significantly higher compared to participants with no history of BTI. Regarding humoral immunity, higher levels were observed with four doses compared to three doses; however, no significant difference was found in cellular immunity. Comparing four doses to five doses, no significant differences were noted in either humoral or cellular immunity. The use of a bivalent vaccine induced a higher humoral immune response compared to a monovalent vaccine, whereas it had no significant impact on cellular immunity. Additionally, individuals who received mRNA-1273 showed significantly higher Log_10_RBD-IgG levels than those who received only BNT162b2.

**Figure 2:**
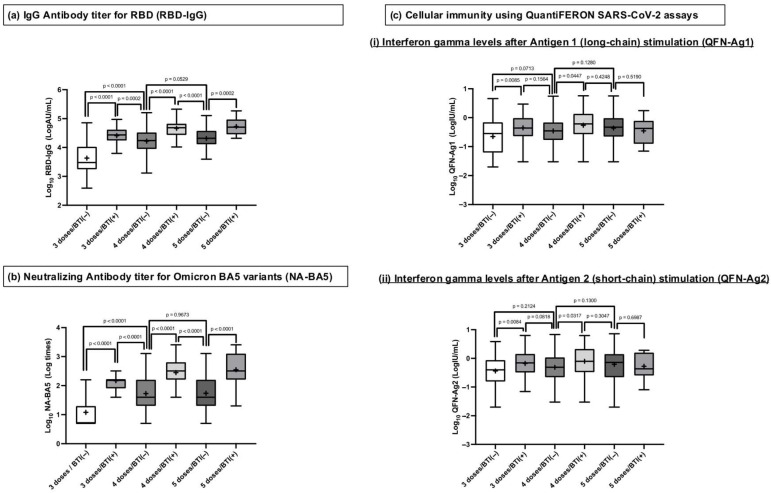
Humoral and cellular immunity at approximately 1 year after the third dose in six groups categorized according to vaccine doses and BTI. The bars show the medians of the logarithms of RBD-IgG, NA-BA5, and QFN-Ag1 and -Ag2 (Log_10_RBD-IgG, Log_10_NA-BA5, Log_10_QFN-Ag1, and Log_10_QFN-Ag2); the boxes show the quartile range, and the error bars show the maximum and minimum after the exclusion of outliers. “+” signs demonstrate the mean of Log_10_RBD-IgG, Log_10_NA-BA5, Log_10_QFN-Ag1, and Log_10_QFN-Ag2. Log_10_RBD-IgG, Log_10_NA-BA5, Log_10_QFN-Ag1, and Log_10_QFN-Ag2 were compared between six groups as follows: 3 doses without BTI (3 doses/BTI(−)), 3 doses with BTI (3 doses/BTI(+)), 4 doses without BTI (4 doses/BTI(−)), 4 doses with BTI (4 doses/BTI(+)), 5 doses without BTI (5 doses/BTI(−)), and 5 doses with BTI (5 doses/BTI(+)). Abbreviations: BTI, breakthrough infections after third-dose administration; RBD-IgG, IgG antibody titer specific to the receptor-binding domain of SARS-CoV-2 measured by chemiluminescent immunoassay; NA-BA5, neutralizing antibody titer measured using the authentic SARS-CoV-2 Omicron BA. Five variants: QFN-Ag1 and -Ag2; interferon gamma levels were measured using QuantiFERON SARS-CoV-2 assays. -Ag1 represents long-chain protein (Antigen 1)-stimulated results, and -Ag2 represents short-chain protein (Antigen 2)-stimulated results.

The levels of Log_10_RBD-IgG, Log_10_NA-BA5, Log_10_QFN-Ag1, and Log_10_QFN-Ag2 for each group are shown in Figure 2. For both Log_10_RBD-IgG and Log_10_NA-BA5, groups with BTI had significantly higher levels compared to groups with the same number of doses without BTI. Additionally, when comparing groups without BTI, those with four doses exhibited higher levels of Log_10_RBD-IgG and Log_10_NA-BA5 than those with three doses, while no significant difference was observed between groups with four and five doses. Furthermore, the group with 3 doses/BTI(+) showed higher levels of Log_10_RBD-IgG and Log_10_NA-BA5 than the group with 4 doses/BTI(−). A similar trend was observed when comparing 4 doses/BTI(+) and 5 doses/BTI(−).

In contrast, for cellular immunity, the 3 doses/BTI(+) group exhibited higher levels of Log_10_QFN-Ag1 and Log_10_QFN-Ag2 than 3 doses/BTI(−), and a similar trend was observed when comparing 4 doses/BTI(+) and 4 doses/BTI(−), but no significant differences were found among the other groups. Although the median values for 3 doses/BTI(+) and 4 doses/BTI(−), as well as 4 doses/BTI(+) and 5 doses/BTI(−), tended to be higher in the BTI(+) groups compared to the BTI(−) groups, these differences were not statistically significant.

**Figure 3:**
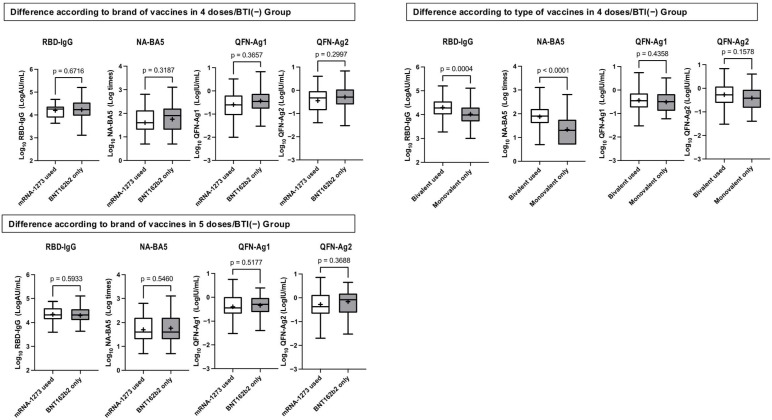
Relationship between the use of the mRNA-1273 vaccine or bivalent vaccines and the levels of Log_10_RBD-IgG, Log_10_NA-BA5, Log_10_QFN-Ag1, and Log_10_QFN-Ag2 among participants who received four or five doses without BTI. The bars show the medians of the logarithms of RBD-IgG, NA-BA5, and QFN-Ag1, and -Ag2 (Log_10_RBD-IgG, Log_10_NA-BA5, Log_10_QFN-Ag1 and Log_10_QFN-Ag2); the boxes show the quartile range; and the error bars show the maximum and minimum values after the exclusion of outliers. “+” signs indicate the mean of Log_10_RBD-IgG, Log_10_NA-BA5, Log_10_QFN-Ag1, and Log_10_QFN-Ag2. Log_10_RBD-IgG, Log_10_NA-BA5, Log_10_QFN-Ag1, and Log_10_QFN-Ag2 were compared based on type, and brand in 4 doses/BTI(−) groups and 5 doses/BTI(−) groups. Since all participants in the five-dose group received the bivalent vaccine, no comparisons were made for this group. Abbreviations: BTI, breakthrough infections after third-dose administration; RBD-IgG, IgG antibody titer specific to the receptor-binding domain of SARS-CoV-2 measured by chemiluminescent immunoassay; NA-BA5, neutralizing antibody titer measured using the authentic SARS-CoV-2 Omicron BA. Five variants: QFN-Ag1 and -Ag2; interferon gamma levels were measured using QuantiFERON SARS-CoV-2 assays. -Ag1 represents long-chain protein (Antigen 1)-stimulated results, and -Ag2 represents short-chain protein (Antigen 2)-stimulated results.

Figure 3 illustrates the relationship between the use of the mRNA-1273 vaccine or bivalent vaccines and the levels of Log_10_RBD-IgG, Log_10_NA-BA5, Log_10_QFN-Ag1, and Log_10_QFN-Ag2 among participants who received four or five doses without BTI. No significant differences in humoral or cellular immunity were observed between those who used the mRNA-1273 vaccine and those who only received the BNT162b2 vaccine. However, the use of the bivalent vaccine in the four-dose group resulted in significantly higher antibody levels compared to the use of the monovalent vaccine alone, while no differences were noted in cellular immunity levels. Since all participants in the five-dose group received the bivalent vaccine, no comparisons were made for this group.

**Figure 4:**
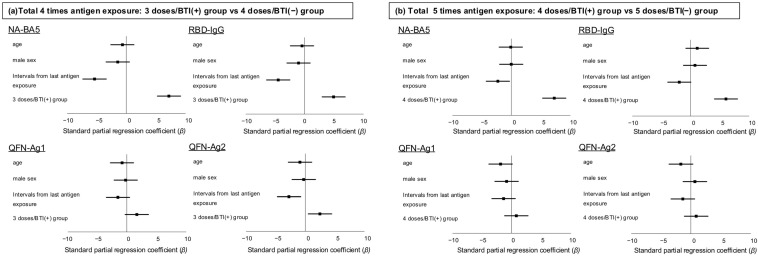
Factors associated with humoral and cellular immunity in participants with a total of four or five antigen exposures. To evaluate whether BTI episodes conferred immunity comparable to that of vaccination, independent of age, sex, and the time since the last antigen exposure, we performed ANCOVA model analyses on the logarithms of RBD-IgG, NA-BA5, and FN-Ag1 and -Ag2 (Log_10_RBD-IgG, Log_10_NA-BA5, Log_10_QFN-Ag1, and Log_10_QFN-Ag2) in participants with (**a**) 3 doses/BTI(+) versus those with 4 doses/BTI(−) and (**b**) 4 doses/BTI(+) versus those with 5 doses/BTI(−). These models incorporated two continuous variables: age and intervals from the last antigen exposure (duration between the sample collection date and BTI episode in BTI(+) groups or the last dose vaccine administration in BTI(−) groups), as well as two categorical variables: sex and group (3 doses/BTI(+) vs. 4 doses/BTI(−) or 4 doses/BTI(+) vs. 5 doses/BTI(−)). Abbreviations: BTI, breakthrough infections after third-dose administration; RBD-IgG, IgG antibody titer specific to the receptor-binding domain of SARS-CoV-2 measured by chemiluminescent immunoassay; NA-BA5, neutralizing antibody titer measured using the authentic SARS-CoV-2 Omicron BA. Five variants: QFN-Ag1 and -Ag2; interferon gamma levels were measured using QuantiFERON SARS-CoV-2 assays. -Ag1 represents long-chain protein (Antigen 1)-stimulated results, and -Ag2 represents short-chain protein (Antigen 2)-stimulated results.

Figure 4 presents the results of an ANCOVA model that includes variables such as age, sex, interval from the last antigen exposure, and group differences (whether the last antigen exposure was a vaccine or BTI), assuming one vaccine dose and one BTI were equivalent to one antigen exposure. For four antigen exposures, a shorter interval from the last antigen exposure and having the last antigen exposure as a BTI were independently associated with higher levels of Log_10_RBD-IgG, Log_10_NA-BA5, and Log_10_QFN-Ag2. For five antigen exposures, having the last antigen exposure as a BTI was independently associated with higher levels of Log_10_RBD-IgG and Log_10_NA-BA5. A shorter interval from the last antigen exposure was associated only with higher levels of Log_10_NA-BA5.

## 4. Discussion

In this follow-up study, we evaluated the impact of BTI and subsequent fourth and fifth booster doses on antibody levels and cellular immunity approximately one year after the third dose of the BNT162b2 vaccine among healthcare workers who had received three doses and had not experienced a BTI before the Omicron variant surge. The results indicated that BTIs were associated with the acquisition of antibody titers, including neutralizing antibodies, which were comparative to the fourth booster dose. Our findings support previous studies indicating that BTIs can induce humoral immunity [11,12,13]. Moreover, in our study, participants who received three vaccine doses and experienced BTIs exhibited higher levels of cellular immunity compared to those without BTIs. This finding is consistent with the results of a cohort study involving 356 healthcare workers in the United States, who received three doses of the BNT162b2 vaccine or two doses of BNT162b2 vaccine and one dose of mRNA-1273 [14].

Furthermore, when considering BTI and vaccinations as individual antigen exposures, regardless of age, sex, or time since the last antigen exposure, BTIs led to higher antibody titers compared to booster doses for the same number of antigen exposures. This result is consistent with other studies. For example, Cohen et al. reported that BTIs after a third dose elicited a more robust humoral immune response than those occurring after a fourth dose in patients with cancer [15]. Furthermore, a cohort study by Bjørlykke et al. involving immunocompromised patients reported that those who contracted the Omicron variant after receiving a third vaccine dose exhibited higher antibody titers compared to those who received four vaccine doses [16].

Srivastava et al. reported that BTIs increased antibodies to a similar titer as that achieved after an additional vaccine dose in naive individuals [7]. Additionally, Campanella et al. reported that in patients with chronic lymphocytic leukemia, a booster dose elicited a humoral immune response similar to that observed after BTIs [17]. These findings suggests that BTIs have at least an equivalent, if not superior, long-term effect on inducing humoral immunity compared to booster doses.

Regarding cellular immunity to the S protein, as assessed by IFN-γ production, BTIs and booster doses exhibited almost equivalent effects when comparing the same number of antigen exposures. Although studies examining cellular immunity are limited, Almazanzar et al. showed no significant difference in cellular immunity 24 weeks after a BTI or a booster dose in individuals who had completed the primary vaccination series [18].

From these results, it can be inferred that in healthy adults who have received at least three doses of the BNT162b2 vaccine, subsequent BTI episodes induce higher humoral immunity and equivalent cellular immunity to the S protein compared to booster doses. Therefore, in terms of immune responses to the S protein, BTIs can be considered antigen exposures that are equivalent to booster doses. In adults who have experienced a BTI, similar immune responses to those following a booster dose are induced, suggesting that the timing of the next booster dose can be planned based on the natural waning of immunity during the time course after last antigen exposure.

One of the major strengths of this study lies in its comprehensive evaluation of both humoral and cellular immunity, specifically assessing not only antibodies against the S protein but also T-cell responses using IFN-γ as a marker. Host defense mechanisms induced by COVID-19 vaccination involve four key components: neutralizing antibodies, memory B cells, CD4+ T cells, and CD8+ T cells. While antibodies play a critical role in neutralizing the virus, T cells contribute to the elimination of infected cells. Therefore, evaluating either humoral or cellular immunity alone is insufficient; both aspects must be assessed to fully understand vaccine-induced protection [19,20]. Moreover, T-cell responses are considered relatively resistant to changes in viral antigenicity caused by mutations [21]. From this perspective, although the assessment of antibodies—which directly mediate viral neutralization—is undoubtedly important, it is equally critical to evaluate T-cell-mediated immunity. This study is, therefore, valuable in that it addresses both dimensions of the immune response.

This study had three limitations. First, the episodes of BTIs were based on self-reported information and medical records based on diagnostic test results. Therefore, subclinical BTI episodes in participants who did not seek medical attention might have been missed. However, during the study period, Keio University, with which participants were affiliated, were mandated to undergo diagnostic testing even when presenting very mild symptoms or following a brief unprotected contact with COVID-19 patients. In this context, the number of subclinical BTIs was relatively low. Second, all immune responses evaluated in this study were limited to those against the S protein. In particular, cellular immunity was assessed solely based on IFN-γ production. Therefore, it remains unclear whether the conclusions would hold if cytotoxic T-cell responses or cellular immunity to other viral antigens were evaluated. Finally, our study participants mainly consisted of healthy adults, limiting the generalizability of the findings to other populations such as children, older individuals, and individuals with compromised immune systems. Future research should aim to include a more diverse set of participants to obtain a more comprehensive understanding of how our findings may apply to these specific populations.

## 5. Conclusions

BTIs occurring after a primary series of COVID-19 vaccinations (first to third dose) induced humoral immunity that is greater than that induced by booster doses (fourth or fifth dose) and elicit cellular immunity comparable to that of booster doses. Therefore, BTIs can be considered antigen exposures that are equivalent to booster doses.

## Figures and Tables

**Table 1:** Demographics of the study participants.

Cumulative Doses	BTI	(n)	Age, Years (Median)	(IQR)	Sex (Male/Female)	(%)	Bivalent Vaccine Used	(%)	mRNA-1273 Used	(%)	Intervals from Last Dose *, Days (Median)	(IQR)	Intervals from BTI **, Days (Median)	(IQR)
ALL	(Any)	429	47	(39.5–54)	114/315	(26.6/73.4)	233	(54.3)	73	(17.0)	105	(60.5–401)	–	–
	BTI+	129	45	(38–51.5)	26/103	(20.1/79.8)	44	(34.1)	19	(14.7)	398	(76–403)	163	(63.5–197)
	BTI−	300	49	(41–55)	88/212	(29.3/70.7)	189	(63.0)	54	(18.0)	82	(57–208.5)	–	–
3	(Any)	146	44	(34.75–50.25)	39/107	(26.7/73.3)	–	–	–	–	402	(400.75–405)	–	–
	BTI+	72	43.5	(37–50)	13/59	(18.1/81.9)	–	–	–	–	402	(401–405)	173	(79.25–203.5)
	BTI−	74	44.5	(31–52)	26/48	(35.1/64.9)	–	–	–	–	402.5	(400–405)	–	–
4	(Any)	179	47	(39–53)	44/135	(24.6/75.4)	129	(72.1)	29	(16.2)	81	(59–143)	–	–
	BTI+	43	43	(38–51)	11/32	(25.5/74.4)	30	(69.8)	9	(20.9)	87	(58–150)	84	(52–195)
	BTI−	136	48	(40.25–54)	33/103	(24.2/75.7)	99	(72.8)	20	(14.7)	80.5	(59.5–139.5)	–	–
5	(Any)	104	54	(47–60)	31/73	(29.8/70.5)	104	(100)	44	(42.3)	57.5	(44.25–68.75)	–	
	BTI+	14	56	(50.5–61.25)	2/12	(14.3/85.7)	14	(100)	10	(71.4)	56.5	(47.75–66)	156	(50–235.5)
	BTI−	90	54	(45.75–60)	29/61	(32.2/67.8)	90	(100)	34	(37.8)	57.5	(44–69)	–	–

* Elapsed days from the last vaccination to sampling. ** Elapsed days from the date of BTI onset to sampling. Abbreviations: BTI, breakthrough infection after the third dose administration; IQR, interquartile range.

## Data Availability

The datasets generated and/or analyzed during the current study are available from the corresponding author on reasonable request.

## References

[1] Polack P.F., Thomas J.S., Kitchin N., Absalon J., Gurtman A., Lockhart S., Perez L.J., Marc P.G., Moreira D.E., Zerbini C. (2020). Safety and Efficacy of the BNT162b2 mRNA Covid-19 Vaccine. N. Engl. J. Med..

[2] Baden R.L., Sahly M.E.H., Essink B., Kotloff K., Fre S., Novak R., Diemert D., Spector A.S., Rouphael N., Creech B.C. (2021). Efficacy and Safety of the mRNA-1273 SARS-CoV-2 Vaccine. N. Engl. J. Med..

[3] Andrews N., Stowe J., Kirsebom F., Toffa S., Rickeard T., Gallagher E., Gower C., Kall M., Groves N., O’Connell M.A. (2022). Covid-19 Vaccine Effectiveness against the Omicron (B.1.1.529) Variant. N. Engl. J. Med..

[4] Bohnert A., Kumbier K., Rowneki M., Gupta A., Bajema K., Hynes M.D., Viglianti E., O’Hare M.A., Osborne T., Boyko J.E. (2023). Adverse outcomes of SARS-CoV-2 infection with delta and omicron variants in vaccinated versus unvaccinated US veterans: Retrospective cohort study. BMJ.

[5] Winokur P., Gayed J., Fitz-Patrick D., Thomas J.S., Diya O., Lockhart S., Xu X., Zhang Y., Bangad V., Schwartz I.H. (2023). Bivalent Omicron BA.1-Adapted BNT162b2 Booster in Adults Older than 55 Years. N. Engl. J. Med..

[6] Martinelli S., Pascucci D., Laurenti P. (2023). Humoral response after a fourth dose of SARS-CoV-2 vaccine in immunocompromised patients. Results of a systematic review. Front. Public Health.

[7] Srivastava K., Carreño M.J., Gleason C., Monahan B., Singh G., Abbad A., Tcheou J., Raskin A., Kleiner G., Bakel V.H. (2024). SARS-CoV-2-infection- and vaccine-induced antibody responses are long lasting with an initial waning phase followed by a stabilization phase. Immunity.

[8] Uwamino Y., Kurafuji T., Takato K., Sakai A., Tanabe A., Noguchi M., Yatabe Y., Arai T., Ohno A., Tomita Y. (2022). Dynamics of antibody titers and cellular immunity among Japanese healthcare workers during the 6 months after receiving two doses of BNT162b2 mRNA vaccine. Vaccine.

[9] Our_World_in_Data. COVID-19 Data Explorer. https://ourworldindata.org/explorers/covid.

[10] Uwamino Y., Yokoyama T., Shimura T., Nishimura T., Sato Y., Wakui M., Kosaki K., Hasegawa N., Murata M. (2022). The effect of the E484K mutation of SARS-CoV-2 on the neutralizing activity of antibodies from BNT162b2 vaccinated individuals. Vaccine.

[11] Walls C.A., Sprouse R.K., Bowen E.J., Joshi A., Franko N., Navarro J.M., Stewart C., Cameroni E., McCallum M., Goecker A.E. (2022). SARS-CoV-2 breakthrough infections elicit potent, broad, and durable neutralizing antibody responses. Cell.

[12] Curlin E.M., Bates A.T., Guzman G., Schoen D., McBride K.S., Carpenter D.S., Tafesse G.F. (2022). Omicron neutralizing antibody response following booster vaccination compared with breakthrough infection. Medicle.

[13] Bates A.T., McBride K.S., Leier C.H., Guzman G., Lyski L.Z., Schoen D., Winders B., Lee Y.J., Lee X.D., Messer B.W. (2022). Vaccination before or after SARS-CoV-2 infection leads to robust humoral response and antibodies that effectively neutralize variants. Sci. Immunol..

[14] Wagenhäuser I., Almanzar G., Förg B.F., Stein A., Eiter I., Reusch J., Mees J., Herzog A., Vogel U., Frey A. (2024). Heterologous and homologous COVID-19 mRNA vaccination schemes for induction of basic immunity show similar immunogenicity regarding long-term spike-specific cellular immunity in healthcare workers. Vaccine.

[15] Cohen I., Campisi-Pfinto S., Rozenberg O., Colodner R., Bar-Sela G. (2023). The Humoral Response of Patients With Cancer to Breakthrough COVID-19 Infection or the Fourth BNT162b2 Vaccine Dose. Oncologist.

[16] Bjørlykke H.K., Ørbo S.H., Tveter T.A., Jyssum I., Sexton J., Tran T.T., Christensen E.I., Kro B.G., Kvien K.T., Jahnsen J. (2023). Four SARS-CoV-2 vaccine doses or hybrid immunity in patients on immunosuppressive therapies: A Norwegian cohort study. Lancet Rheumatol..

[17] Campanella A., Capasso A., Heltai S., Taccetti C., Albi E., Herishanu Y., Haggenburg S., Chatzikonstantinou T., Doubek M., Kättström M. (2024). Additional booster doses in patients with chronic lymphocytic leukemia induce humoral and cellular immune responses to SARS-CoV-2 similar to natural infection regardless ongoing treatments: A study by ERIC, the European Research Initiative on CLL. Am. J. Hematol..

[18] Almanzar G., Koosha K., Vogt T., Stein A., Ziegler L., Asam C., Weps M., Schwägerl V., Richter L., Hepp N. (2024). Hybrid immunity by two COVID-19 mRNA vaccinations and one breakthrough infection provides a robust and balanced cellular immune response as basic immunity against severe acute respiratory syndrome coronavirus 2. J. Med. Virol..

[19] Sadarangani M., Marchant A., Kollman T.R. (2021). Immunological mechanisms of vaccine-induced protection against COVID-19 in humans. Nat. Rev. Immunol..

[20] Sette A., Crotty S. (2021). Adaptive immunity to SARS-CoV-2 and COVID-19. Cell.

[21] Tarke A., Sidney J., Methot N., Yu D.E., Zhang Y., Dan M.J., Goodwin B., Rubiro P., Sutherland A., Wang E. (2021). Impact of SARS-CoV-2 variants on the total CD4+ and CD8+ T cell reactivity in infected or vaccinated individuals. Cell Rep. Med..

